# Reports on the Hospitals of the United Kingdom

**Published:** 1910-06-11

**Authors:** Henry Burdett


### REPORTS

ON THE

### Hospitals of the United Kingdom.

By SIR HENRY BURDETT, K.C.B., K.C.V.O.

### L.—LEEDS UNION INFIRMARY.

This Infirmary was established in 1879, and contains 900 beds, of which 850 are on an average constantly occupied. It is a new Infirmary on the pavilion principle, on which a large sum has evidently been expended. Glazed bricks and tiles have been extensively used for the interior walls, and the general effect is good. The appurtenances of the wards and the fittings are above the average, but the architect has evidently not had much, if any, experience of hospital buildings, and the ward units are in consequence neither complete nor as convenient and perfect as they might have been, having regard to the date of the buildings. Further, the institution has no operation theatre and has no visiting surgeon or visiting medical staff.

### Condition of the Wards.

The condition of the wards is on the whole satisfactory. They present a pleasant and attractive appearance, and the furniture is of good design, though the workmanship or the newness of the material used, or both, have caused several articles to exhibit signs of wear and tear which they ought not to do. We presume the Guardians will insist on the contractors putting all such matters right. Otherwise the bedsteads, tables, drawers, and other furniture present a smart appearance, and are better than those to be met with in most Poor-law Infirmaries. Unfortunately the medical superintendent, Dr. Allan, was away ill, and the assistant superintendent was out when we visited the Infirmary, but we were courteously received by the acting assistant medical officer, who took a good deal of trouble and showed great interest in his work.

There can be no doubt that it is wrong for a Board of Guardians in the present day to build a great Poor-law Infirmary like this and not to provide an operation theatre. A number of poor people requiring operations necessarily find their way into the wards of the Poor-law hospitals, where they ought to have the best of treatment and to be guaranteed that, whatever the nature of their case, it will be promptly treated and dealt with in the best possible manner. On the whole, experience would seem to teach that a modern Poor-law Infirmary to be up to date and efficient should be placed under the management of a medical superintendent, who should have the co-operation and help of a visiting surgeon of repute, with every facility provided for surgical work.

The absence of these necessary adjuncts renders the Leeds Poor-law Infirmary very deficient, and it would be interesting to know whether, in the circumstances, a patient may not have a cause of action against the Guardians, individually and collectively, should a case occur where provable harm results from the absence of an adequate surgical provision and staff.

### The Food Supplies.

Another point which came under our notice was the quality of the food. The contractors had recently been changed, and on several occasions, including the day of our visit, the meat, or some of it, had been distinctly bad. Remonstrances addressed to the steward had not resulted in better supplies, and it was plainly stated that the reason might be that the steward was afraid of offending the contractor or some of the Guardians who might uphold him. We were also informed that other supplies, including the eggs, under the existing contracts, were unsatisfactory. Of all places, a workhouse is one where the quality of the food supplied should be of the very best. The inmates are absolutely defenceless, and it should be the pride, as it is undoubtedly the duty, of every official and every Guardian to insist that nothing that is not of the best should be received at these Infirmaries or issued to the inmates. The consequence of supplying bad meat, the quality of which may in a measure be concealed by the cooking, is a very grave one. This is the first occasion on which we have met with complaints of the existence of abuses of this kind, and we hope that the people of Leeds and the Guardians will take steps to provide that nothing of this nature will be suffered at this Infirmary.

The condition of the wards and the state of order which was everywhere apparent rather astonished us, having regard to the relative smallness of the nursing staff. Indeed, it is a mystery how so few people can maintain such high efficiency as these wards exhibited on a close inspection. There are a large number of children in the children's wards, where a larger staff is especially necessary and should be provided. The present medical staff provision is short; the absence of an operation theatre is regrettable; and the result must be anything but satisfactory from the patients' point of view, and consequently from the point of view of a resident medical officer. At least, that is our view, but we were not able to confirm it for the reasons we have stated.

### The Nurses' Home.

We published a plan of this home in The Hospital of November 5, 1894, page 86. The bedrooms are comfortable, but it is a bad system which entails the nurses having to go from their bedrooms to the lavatory in order to wash in the morning, especially as the number of basins provided, having regard to the number of nurses requiring to use them, is often too small. The nursing staff consists of a matron, Miss Gittins, whose ability is proved by the condition of the wards and the efficiency of the nursing staff: three assistants, including the home sister and night superintendent, twelve charge nurses, and fifty-eight nurses and probationers. There are also four male and five female attendants. The training school was established here in 1894, with a three years' course, and certificates are granted.

Having regard to the size of the staff, we are of opinion that the nurses' sitting-rooms are too small, and we also consider that it is desirable that each nurse's bedroom should contain a washstand, jug, basin, and water-supply. The home would also be much more complete if a room or rooms were provided where the nurses might read or write in quietude. The storage accommodation for boxes, the absence of a proper provision of downstair lavatories and a boot-room, and other minor but important matters of this kind, point to the fact that the plan was designed by some one who has not had a large experience in the planning of this type of building so that it may fully meet the requirements of modern nursing.

General Comments.

We should say, generally, that it is fair to the Guardians to conclude from the large expenditure upon the buildings that they had every desire to provide a modern hospital. If this be a correct view, it is a thousand pities that they did not call in expert advice, so that when they were about it they might have made everything as perfect as possible, and avoided the blot of omitting the provision of an operation theatre unit. That unit should be supplied without delay, and a visiting surgeon of experience appointed. There seems, too, to be reason to look into the whole of the provisioning arrangements and the enforcement of the terms of various contracts, so as to provide that in no case shall any food be accepted which is not fit for use, or which is bad, or which is indeed anything but of the best quality. Where bad food is permitted to enter an establishment of this kind there must be some reason why the contractors are not kept strictly to the terms of their contract, and for this reason no member of the Board of Guardians can be held to discharge properly his duties, or to live up to his responsibilities, who tolerates any such condition of affairs as was pointed out on the day of our inspection of this Infirmary.

---

The **Royal Alexandra Hospital for Sick Children, Brighton,** will be reported on next week.

### SOME AUSTRALIAN NOTES.

FROM A SPECIAL CORRESPONDENT.

### THE HOSPITALS.

The hospitals of Melbourne are all gradually attempting to establish an advisory board for appointing the medical and surgical staffs, the latest to fall into line being the Women's Hospital, whose contributors and life governors held a special meeting on March 11 last, and adopted a scheme similar to that passed by the Melbourne Hospital Committee some years ago.

The trustees of the Edward Wilson estate, which is contributing £120,000, have approved of the plans for the new Melbourne Hospital, and work will be proceeded with at an early date.

St. Vincent's Hospital, now a recognised clinical school, was the scene of a great gathering on the afternoon of March 19 last, when the new pathological wing for clinical teaching was formally declared ready for use. A lady, Mrs. John Southall, provided the whole of the necessary funds—£1,200. Sir John Madden, Chief Justice of Victoria, performed the opening ceremony, and Mrs. Southall unveiled the commemoration tablet and, on behalf of the Sisters of Charity, presented Sir John with a gold key as a souvenir.

The State Treasurer (Mr. Watt), with the Under-Treasurer (Mr. Minogue), and the Inspector of Charities (Mr. Meek), spent a short holiday recently in scrambling about the mountainous country in the east of the State. The party found the Wood's Point (Goldfield) hospital in an extremely dilapidated condition, and the Walhalla (Goldfield) hospital, new but short of funds. Mr. Watt promised State help in both cases.

### SCHOOL CHILDREN'S HEALTH.

The three medical officers appointed to examine school children, Drs. Harvey Sutton, Mary Booth, and Jean Greig, began work early in February with a (State) school of 550, at Port Melbourne. The first was a great field day for the children. In the office Dr. Sutton took the boys four and five at a time. In a classroom the ladies were inspecting the girls, and in a shelter-shed four members of the Odontological Society were examining boys' and girls' teeth.

The medical officers spent three weeks at Port Melbourne, and their report was that the scholars were an unusually clean and healthy lot—perhaps owing to the proximity to the sea. They are now engaged on a school several miles inland. Cards with about twenty questions are sent to the parents to be filled up, which is generally done in an intelligent and business-like manner, and the children are measured, weighed, tested for sight and hearing, etc., and where they show constitutional weakness, the parents are recommended to seek medical advice.

Dr. J. S. Elkington, Chief Commissioner for Public Health, Queensland, is commencing similar examinations of school children in his State. In the western part of Queensland a number of schools have been fitted with wire-netting doors and windows, and the protection afforded to the children is reported to be much appreciated.

### INEBRIATES.

Until the other day no home for women drunkards existed in Victoria—only within a few years have homes for men been provided. Now the want is supplied by the Salvation Army. The Home stands on five acres of land in East Malvern, a suburb of Melbourne, and will accommodate 37 patients. All classes of female inebriates will be treated, and fees will be charged according to the patients' means. The Government, it is understood, has something of the kind in view, but meantime patients will be sent to this Home, and paid for.

